# Herbal Combination of *Angelica gigas*, *Zingiber officinale*, and *Aconitum carmichaeli* Alleviates High Fat Diet‐Induced Non‐Alcoholic Fatty Liver Disease in Mice Through NRF2‐Mediated Regulation of Adipogenesis and Non‐Shivering Thermogenesis

**DOI:** 10.1002/fsn3.71199

**Published:** 2025-11-25

**Authors:** Harim Kim, Hye‐Lin Kim, Mina Boo, Hyunyoung Choi, Jaehyun Han, Seunghyun Nam, So Jung Kang, Jae Kyeom Kim, Yohan Han, Ji Hoon Jung, Woojin Kim, Kwan‐Il Kim, Jae‐Young Um, Jinbong Park, Leo E. Otterbein, Hyo In Kim, Seong‐Gyu Ko

**Affiliations:** ^1^ Department of Science in Korean Medicine, Graduate School Kyung Hee University Seoul Republic of Korea; ^2^ College of Korean Medicine Kyung Hee University Seoul Republic of Korea; ^3^ Department of Clinical Korean Medicine, Graduate School Kyung Hee University Seoul Republic of Korea; ^4^ Department of Food and Biotechnology Korea University Sejong Republic of Korea; ^5^ Institute of Wonkwang Medical Science and Department of Microbiology Wonkwang University School of Medicine Iksan Republic of Korea; ^6^ Korean Medicine‐Based Drug Repositioning Cancer Research Center Kyung Hee University Seoul Republic of Korea; ^7^ Korean Medicine Digital Convergence Center (KMDC) Kyung Hee University Seoul Republic of Korea; ^8^ Department of Surgery, Beth Israel Deaconess Medical Center Harvard Medical School Boston Massachusetts USA

**Keywords:** adipogenesis, gut microbiome, herbal medicine, non‐shivering thermogenesis, obesity, oxidative stress

## Abstract

Obesity is a complex metabolic disorder characterized by excessive fat accumulation and is closely associated with non‐alcoholic fatty liver disease (NAFLD), a condition that increases the risk of metabolic complications such as insulin resistance, type 2 diabetes, and cardiovascular diseases. JI017 is a recently optimized multi‐herbal formula composed of *Angelica gigas*, *Zingiber officinale* , and processed *Aconitum carmichaeli*. Clinical records of these three herbs indicate their potentially synergistic anti‐obesity effects. This study was conducted to verify the effects of JI017 in an animal model of obesity‐associated NAFLD. The effect of JI017 on glucose metabolism, white adipose tissue (WAT) adipogenesis, brown adipose tissue (BAT) thermogenesis, liver oxidative stress, and gut microbiota composition was assessed. The results showed that JI017 improved glucose sensitivity, reduced WAT mass by suppressing PPARγ and C/EBPα expression, and increased BAT thermogenesis through upregulation of UCP1 and PGC‐1α. Additionally, JI017 reduced liver oxidative stress by increasing HO‐1 and NRF2 expression and modulated the gut microbiota by restoring the *Firmicutes*/*Bacteroidetes* ratio. These findings on the multi‐targeted effects of JI017 suggest its potential as a promising therapeutic approach for metabolic diseases including NAFLD.

## Graphical abstract

The multi‐herbal formula JI017, consisting of *Angelica gigas*, *Zingiber officinale* , and *Aconitum carmichaeli,* alleviates high‐fat diet‐induced non‐alcoholic fatty liver disease by activating the NRF2 antioxidant pathway. This central mechanism suppresses adipogenesis in the liver and white adipose tissues while simultaneously enhancing thermogenesis in brown adipose tissue. Concurrently, JI017 modulates the gut microbiome, notably decreasing the *Firmicutes*/*Bacteroidetes* ratio, suggesting a multi‐targeted approach to improving metabolic health.

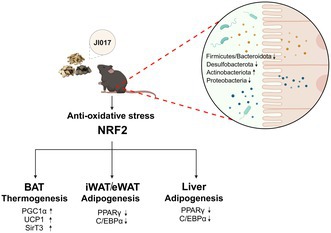

**Abbreviations**
ALT: alanine transaminase
AST: aspartate aminotransferase
AUC: area under the curve
BAT: brown adipose tissue
C/EBPα: CCAAT/enhancer‐binding protein alpha
eWAT: epididymal white adipose tissue
GMP: good manufacturing practice
H&E: hematoxylin and eosin
HFD: high‐fat diet
HO‐1: heme oxygenase‐1
HPLC: high‐performance liquid chromatography
iWAT: inguinal white adipose tissue
LDL: low‐density lipoprotein
NAFLD: non‐alcoholic fatty liver disease
NC: normal control
NRF2: nuclear factor erythroid 2‐related factor 2
OGTT: oral glucose tolerance test
PBS: phosphate‐buffered saline
PPARγ: peroxisome proliferator‐activated receptor gamma
PVDF: polyvinylidene difluoride
ROS: reactive oxygen species
SIRT3: sirtuin 3
TG: triglycerides
UCP1: uncoupling protein 1
WAT: white adipose tissue

## 1 Introduction

Obesity has become a critical global health issue that significantly contributes to the incidence of metabolic disorders such as type 2 diabetes, cardiovascular disease, and non‐alcoholic fatty liver disease (Godoy‐Matos et al. 2020). According to the World Health Organization (WHO), adults with overweight or obesity reached 2.8 billion in 2022 (WHO n.d.). Obesity is characterized by excessive adipose tissue accumulation resulting from an imbalance between energy intake and expenditure. The pathophysiology of obesity is multifaceted, involving hormonal dysregulation, particularly in hormones like insulin, leptin, and ghrelin, which regulate appetite and metabolism (Wen et al. 2022). Obesity is linked to chronic low‐grade inflammation in the adipose tissue which contributes to complications including as insulin resistance and metabolic syndrome (Hildebrandt et al. 2023).

Among the obesity‐related diseases, non‐alcoholic fatty liver disease (NAFLD) has emerged as a major public health concern. NAFLD affects approximately 25%–30% of the global population, with prevalence approaching ~70% among individuals with obesity (Huh et al. 2022). Pathophysiologically, insulin resistance, adipose tissue inflammation, and oxidative stress collectively drive NAFLD progression, eventually increasing the risk of non‐alcoholic steatohepatitis (NASH), fibrosis, cirrhosis, or hepatocellular carcinoma (Younossi et al. 2022). Currently, there is no specific pharmacological therapy approved for NAFLD (An and Sohn 2023). Novel therapies such as GLP‐1 receptor agonists (Targher et al. 2023) and PPAR agonists (K. S. Kim and Lee 2020) have shown promise but remain limited by adverse effects and inconsistent responses. As a result, there is growing interest in alternative therapies, particularly herbal medicines. Herbal formulas have gained interest for their potential to improve metabolic health and provide a more holistic approach to treating obesity (Boix‐Castejón et al. 2023; Park et al. 2022; Roy et al. 2024). Notably, recent studies have revealed that multi‐herbal formulas can offer benefits by targeting multiple metabolic pathways simultaneously, potentially overcoming the limitations of single‐ingredient treatments (Li, Wang, et al. 2023; Li, Wu, et al. 2023; Shi et al. 2022; Zhang et al. 2024).

This study investigates the effect of JI017, a multi‐herbal formula composed of *Angelica gigas*, *Zingiber officinale* , and *Aconitum carmichaeli*, in alleviating high‐fat diet (HFD)–induced obesity and NAFLD in C57BL/6J mice. *Angelica gigas* and *Zingiber officinale* have long been consumed as foods and traditional medicines (Ahn et al. 2023; Garza‐Cadena et al. 2023). *Aconitum carmichaeli* is also used as a food supplement (Chan 2014), but due to its potential toxicity, it is administered only at regulated doses after processing to detoxify (Chan et al. 2021; Zhang et al. 2022). NAFLD emerges from multi‐defects such as excessive hepatic lipid, dysfunction in adipose tissue metabolism, low‐grade inflammation, and barrier‐mediated oxidative stress (Loomba et al. 2021). We therefore hypothesized that JI017, a 2:1:1 ratio formula of these three herbs, would engage complementary control points important in NAFLD.

Previous research indicates that these individual herbal components regulate key factors in metabolic disorders. However, comprehensive evaluation of the combination of these herbs remains limited. Here, we assess its potentially synergistic effect on glucose metabolism, adipogenesis, non‐shivering thermogenesis, and oxidative stress. Understanding how JI017 modulates these pathways may provide insights into its promise as a multi‐target therapeutic approach for NAFLD.

## 2 Material and Methods

### 2.1 Preparation of JI017 Powder

The herbal formula JI017 was composed of *Angelica gigas*, *Zingiber officinale* , and processed *Aconitum carmichaeli* in a 2:1:1 ratio. The ingredients were mixed, soaked in 70% ethanol, and extracted at 80°C for 3 h. The extract was filtered, evaporated, and lyophilized to yield JI017 powder. All procedures were conducted in a good manufacturing practice (GMP) facility at the Hanpoong Pharm and Food Company (Jeonju, Republic of Korea). The powder was stored at −80°C and dissolved in phosphate‐buffered saline (PBS) immediate before use.

### 2.2 High‐Performance Liquid Chromatography (HPLC)

HPLC was performed on a Waters HPLC system comprising a Waters 600S controller, Waters 626 pump, Waters temperature control module, Waters In‐Line degasser, Waters 717 plus autosampler, and a Waters 996 photodiode array detector (PDA). Constituents of JI017 were analyzed by HPLC (ODS C18, 250 × 4.6 mm i.d. 5 μm, 2020 Shimadzu, Kyoto, Japan). Nodakenin, 6‐gingerol, decursin, and aconitine (all purchased from Sigma‐Aldrich, Burlington, MA, USA) served as standards. The column temperature was maintained at 35° *C. mobile* phase was as follows (A: water, B: acetonitrile): 5%–20% B, 0–40 min; 20% B, 40–50 min; 20%–5% B, 50–55 min; 5% B, 55–70 min. The flow rate was maintained at 1.2 mL/min with an injection volume of 10 μL and UV detection absorbance of 208 nm. Chromatograms were processed with Empower pro software, Build 1154 (Waters, Milford, MA, USA).

### 2.3 Network Pharmacology Analysis

Network pharmacology analysis was conducted to identify potential targets of JI017 in obesity and NAFLD. Disease‐related target libraries were constructed using GeneCards (http://genecards.org/) and CTD (https://ctdbase.org/). To reduce false positives, only curated targets directly associated with the diseases were retained, and duplicates were removed. Compound‐associated targets were predicted using the Similarity Ensemble Approach (SEA, https://sea.bkslab.org/) and SuperPred (https://prediction.charite.de/), resulting in 135 targets for Nodakenin, 211 for 6‐Gingerol, and 126 for Decursin. After merging and removing overlapping targets, a total of 332 genes were identified for the JI017 network. Overlapping genes between compound‐ and disease‐associated sets were identified using a Venn diagram (http://www.bioinformatics.com.cn/), and the interacting genes were collected as candidate targets. A protein–protein interaction (PPI) network was constructed using STRING (https://stringdb.org/). Hub genes were ranked by degree centrality using CytoHubba in Cytoscape (v3.9.1 https://cytoscape.org/), and the top 15 were selected.

### 2.4 GO and KEGG Pathway Enrichment Analysis

Gene Ontology (GO) and Kyoto Encyclopedia of Genes and Genomes (KEGG) enrichment analyses were performed using the Database for Annotation, Visualization and Integrated Discovery (DAVID, https://david.ncifcrf.gov/) and an online bioinformatics platform (http://www.bioinformatics.com.cn/). GO categorizes genes into cellular components (CC), molecular functions (MF), and biological processes (BP). KEGG enrichment analysis was used to explore the biological pathways related to key genes. Bubble charts and pathway visualizations for KEGG enrichment and GO terms (CC, MF, BP) were generated on the bioinformatics platform (http://www.bioinformatics.com.cn/).

### 2.5 Molecular Docking Simulation

Molecular docking of the JI017 constituents with target proteins was performed using CB‐Dock2 (https://cadd.labshare.cn/cb‐dock2/index.php). The NRF2–KEAP1 complex (PDB ID: 5WFV) was used as the receptor. The top‐ranked poses were selected based on binding scores. Docking results were visualized using UCSF Chimera (https://www.cgl.ucsf.edu/chimera/). For NRF2–KEAP1, Nodakenin, 6‐Gingerol and Decursin were docked at the protein–protein interface, suggesting its potential to modulate NRF2 signaling by interfering with KEAP1 binding.

### 2.6 Animal Experiment

The animal study protocol was approved by the Institutional Animal Care and Use Committee of Kyung Hee University (approval number: KHSASP‐22‐568, date of approval: Nov 30, 2022). 4‐week‐old male C57BL/6J mice (Daehan Biolink Co., Eumsung, Korea) were maintained for 1 week prior to the experiments for acclimatization. The mice were fed with a 60% kcal high fat diet (HFD) (Rodent diet D12492, Research Diets, New Brunswick, NJ, USA) for 4 weeks to induce obesity and NAFLD, in accordance with our previous reports. Then, mice were divided into two groups (*n* = 5), fed for 6 additional weeks with either (a) HFD alone or (b) HFD plus JI017 (200 mg/kg/day). The treatment dose of JI017 was determined based on our previous reports (Ku et al. 2023; Lee et al. 2021). A group fed a normal chow diet for 10 weeks was used as normal control (NC). Body weight was measured twice per week.

### 2.7 Blood Serum Analysis

Serum total cholesterol (TC), low‐density lipoprotein (LDL) cholesterol, high‐density lipoprotein (HDL) cholesterol, triglyceride (TG), alanine aminotransferase (ALT), aspartate aminotransferase (AST), and creatinine were analyzed using enzymatic colorimetric methods by SLC Healthcare (Seoul, Republic of Korea).

### 2.8 Oral Glucose Tolerance Test (OGTT)

At Week 10, mice were fasted for 16 h, then orally administered glucose (2 g/kg body weight; prepared in distilled water). Blood glucose was measured from the tail via a small distal tail nick (~2 mm) at 0, 10, 20, 40, 90, and 120 min after gavage, using a glucometer (Accu‐Chek Performa, Roche Diagnostics, Mannheim, Germany).

### 2.9 Hematoxylin and Eosin (H&E) Staining

Section preparation and H&E staining of BAT, iWAT, eWAT, and liver tissues were performed as described previously (Kang et al. 2020). Briefly, the tissues were collected, washed in PBS and fixed in 10% formalin. After rinsing, tissue processing was performed, and the tissues were embedded in paraffin. To deparaffinize the tissue sections, they were immersed in xylen I and II for 5 min each, and then dehydrated by immersion in ethanol 100% I, 100% II, 90%, 80%, and 70% for 3 min each. The tissue sections were subsequently stained with H&E and toluidine blue. Stained tissue sections were photographed using an EVOS M7000 microscope (Thermo Fisher Scientific, MA, USA). Skin epithelial thickness was measured using the ImageJ software (National Institute of Health, Bethesda, MD, USA).

### 2.10 Western Blot Analysis

Western blot analyses were conducted using harvested tissues, as described previously (Kang et al. 2020). Proteins were extracted from the tissue using lysis buffer (Cell Signaling Technology, Danvers, MA, USA) and incubated on ice for 30 min. Tissue lysates were then centrifuged at 13,000×*g* at 4°C for 30 min, and the supernatant were collected. After quantifying the lysate using a protein assay reagent (Bio‐Rad Laboratories, Hercules, CA, USA), samples were prepared with equal protein concentrations to ensure uniformity. Sodium dodecyl sulfate (SDS)‐polyacrylamide gel electrophoresis was used to separate the lysates, followed by transfer of the separated proteins onto a polyvinylidene difluoride (PVDF) membrane. The membrane was blocked with 5% skim milk, and the primary antibodies (1:1000) were applied overnight at 4°C. The membranes were then incubated with horseradish peroxidase (HRP)‐conjugated secondary antibodies (1:10,000) at room temperature for 1 h. The protein signals were detected utilizing Amersham ECL Detection Reagents (Cytiva, Wilmington, DE, USA) in a Davinch CAS‐900MF (Davinch‐K, Seoul, Republic of Korea).

### 2.11 16 s Microbiome Analysis

16 s microbiome analysis was performed at Novogene Co. Ltd. Briefly, fecal genomic DNA was extracted from fecal samples with QIAamp DNA Stool Mini Kit (Qiagen, Hilden, Germany) according to the manufacturer's protocol. The DNA concentration and purity were measured using a NanoDrop 2000 spectrophotometer (Thermo Fisher Scientific, Waltham, MA, USA). 16 s rRNA/18 s rRNA/ITS genes of distinct regions (16SV4/16SV3/16SV3‐ V4/16SV4‐ V5, 18SV4/18SV9, ITS1/ITS2, ArcV4) were amplified using barcoded primers. The PCR products were purified using magnetic bead purification. Samples were mixed in equal‐density ratios based on the concentration of PCR products. After thorough mixing, the PCR products were detected and target bands were recovered. Sequencing libraries were generated and indexes were added. The library was quantified using Qubit and real‐time PCR, and size distribution was determined with a bioanalyzer. Quantified libraries were pooled and paired‐end sequenced on a Illumina platform (Illumina, San Dieago, CA, USA) according to the required effective library concentration and data amount required. Paired‐end reads were assigned to samples based on their unique barcode and trimmed by removing the barcode and primer sequences. Paired‐end reads were merged using FLASH (v1.2.11, http://ccb.jhu.edu/software/FLASH/) (Magoč and Salzberg 2011). For the effective tags, denoising was performed with DADA2 or Deblur module in the QIIME2 software (QIIME2‐202202) to obtain initial amplicon sequence variants (ASVs) (default: DADA2). Species annotation was performed using QIIME2. To examine the phylogenetic relationship of each ASV and the differences of the dominant species among different groups, multiple sequence alignment was performed using QIIME2. The absolute abundance of ASVs was normalized using a standard of sequence number corresponding to the sample with the least sequences. Subsequent analysis of alpha diversity was performed based on the output normalized data.

### 2.12 Statistical Analysis

All data are presented as mean ± standard error mean (SEM) of three or more independent experiments. Statistical differences were analyzed by One‐way ANOVA via Prism 8 (GraphPad Software, San Diego, CA, USA). Probability values of *p < 0.05, **p < 0.01, ***p < 0.001, and ****p < 0.0001 were considered statistically significant.

## 3 Results

### 3.1 Network Pharmacology Analysis Identifies Potentially Beneficial Effect of JI017 on Obesity

**FIGURE 1:**
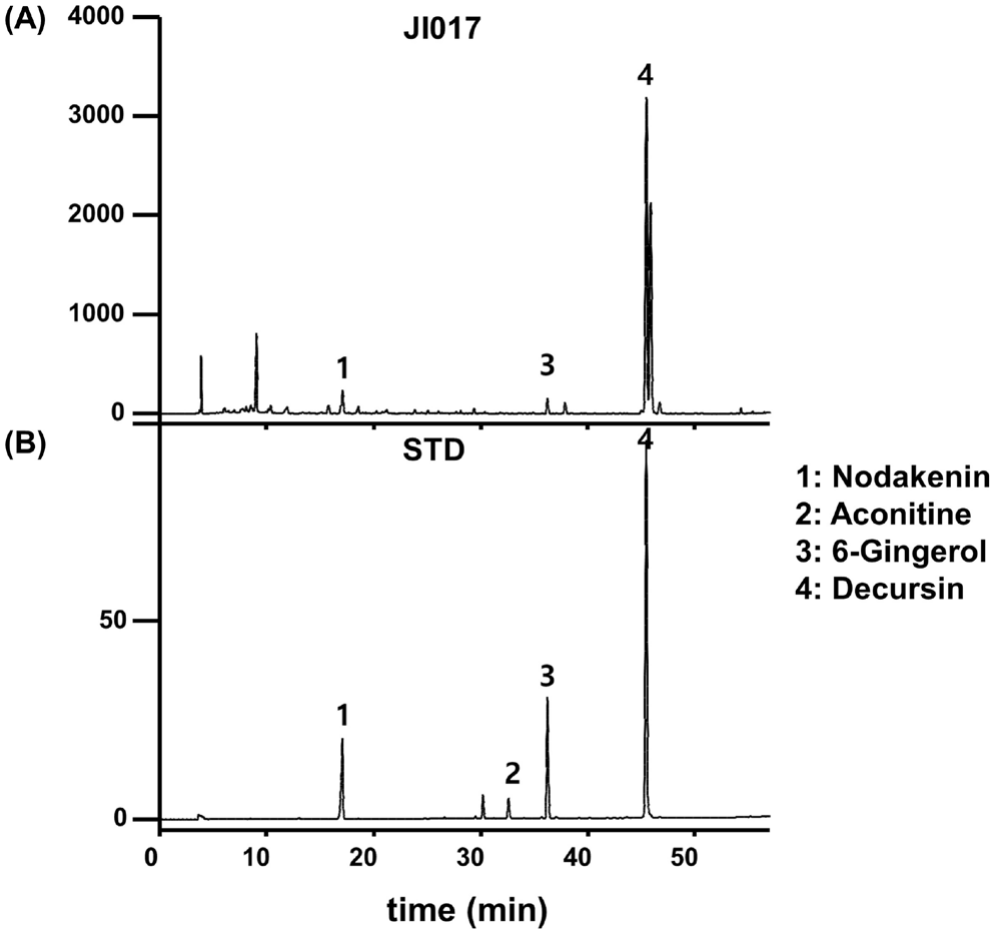
Identification of chemical compositions of JI017. Characterization of JI017 was based on retention times and UV absorption of standard chemicals at 280 nm. HPLC profiles of (A) JI017 and (B) standard mixture of nodakenin, aconitine, 6‐gingerol, and decursin are shown. Nodakenin, aconitine, 6‐gingerol, and decursin were detected at 17, 33, 36, and 46 min, respectively.

**FIGURE 2:**
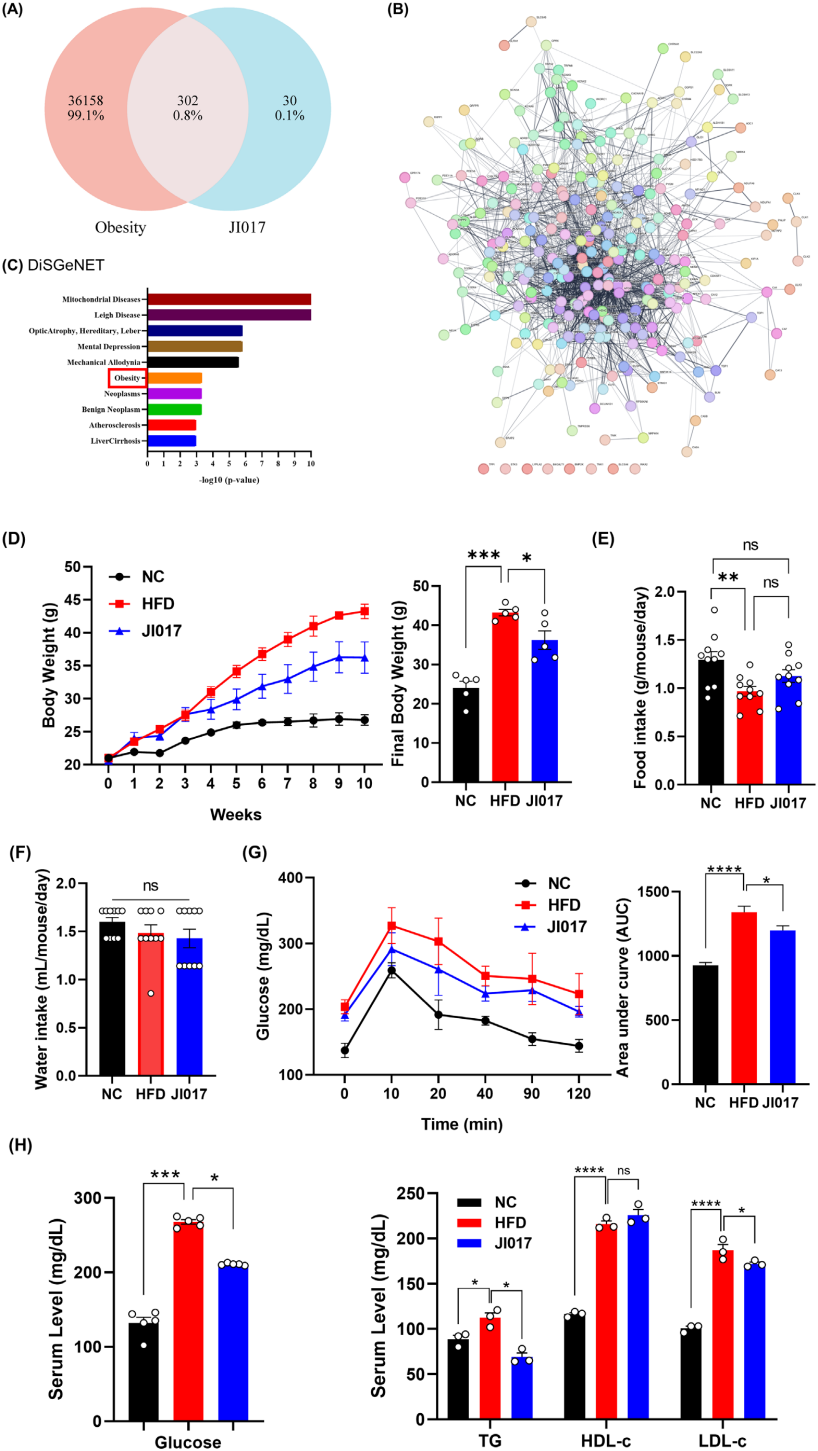
Effect of JI017 on obesity‐related parameters in HFD‐fed C57BL/6J mice. Network pharmacology analysis determines (A) 302 shared genes related to JI017 and obesity. (B) PPI network and (C) DisGeNET analysis of the common genes. (D) Weekly body weight changes and final body weight at the time of sacrifice. (E) Food intake and (F) water consumption were measured. (G) Blood glucose levels at 0, 10, 30, 60, 90, 120 min after glucose challenge were measured and AUC was calculated. (H) Serum levels of glucose, triglycerides, HDL cholesterol, and LDL cholesterol were measured. Significant differences between the groups were assessed using one‐way ANOVA with Tukey's multiple comparison test. Data are expressed as the mean ± SEM of three or more independent experiments. *p < 0.05, **p < 0.01, ***p < 0.001, ****p < 0.0001. NC, normal control group; HFD, high‐fat diet group; JI017, HFD diet plus JI017 group.

HPLC was performed to identify active compounds of JI017 (Figure 1). A mixture of standards of nodakenin and decursin (for *Angelica gigas*), aconitine (for *Aconitum carmichaeli*), and 6‐gingerol (for *Zingiber officinale* ) ws used. Nodakenin, 6‐gingerol, and decursin were detected at 17 min, 36 min, and 46 min, respectively. Aconitine, a compound derived from *Aconitum carmichaeli*, is highly toxic (Chan et al. 2021) and is thus removed by processing; accordingly, it was not detected in the JI017 extract. Intersection analysis of compound‐ and disease‐related gene sets revealed that 302 targets were shared between JI017 and obesity‐associated genes, representing 91% of JI017‐associated targets (Figure 2A). To gain systems‐level insight, we constructed a PPI network using these genes. The resulting network comprised densely interconnected nodes, suggestive of functional clustering and pathway co‐regulation (Figure 2B). To further characterize the disease relevance of JI017 target genes, we performed disease enrichment analysis using the DisGeNET platform. Among the top‐ranked disease associations, obesity was significantly enriched, supporting the rationale for targeting this condition with JI017 (Figure 2C). Collectively, these findings suggest that JI017 may exert therapeutic effects in obesity via modulation of transcriptional regulators and inflammatory cascades, acting on a network of interrelated molecular targets rather than a single axis of intervention.

### 3.2 JI017 Inhibits Body Weight Gain and Improves Glucose Metabolism in HFD‐Fed C57BL/6J Mice

We induced obesity in male C57BL/6J mice by providing ad libitum access to a 60% kcal HFD. After 5 weeks, when the body weight differences reached statistical significant difference between the NC and HFD groups, JI017 was administered via oral gavage at 200 mg/kg/day. After an additional 5 weeks of HFD feeding, the JI017‐treated group showed reduced body weight compared with the HFD‐fed control group (Figure 2D). However, food intake (Figure 2E) and water intake (Figure 2F) did not differ between groups. This suggests that JI017 has a direct effect on body weight regulation, likely mediated by metabolic changes rather than changes in appetite or hydration. An OGTT was conducted to investigate the effect of JI017 on glucose tolerance. JI017‐treated mice exhibited markedly improved glucose tolerance during the OGTT. At 20, 40, 90, and 120 min post‐glucose administration, blood glucose levels were significantly lower in the JI017 group compared to the control group (Figure 2G). Additionally, lipid profiles were significantly improved in the JI017‐treated group, with reduced serum TG and LDL‐c levels, while HDL‐c levels were slightly elevated (Figure 2H). These results indicate that JI017 exerts beneficial effects on both glucose metabolism and lipid regulation, potentially modulating key risk factors for metabolic diseases.

### 3.3 JI017 Suppresses Adipogenesis in WAT Enhances Non‐Shivering Thermogenesis in BAT of HFD‐Fed C57BL/6J Mice

**FIGURE 3:**
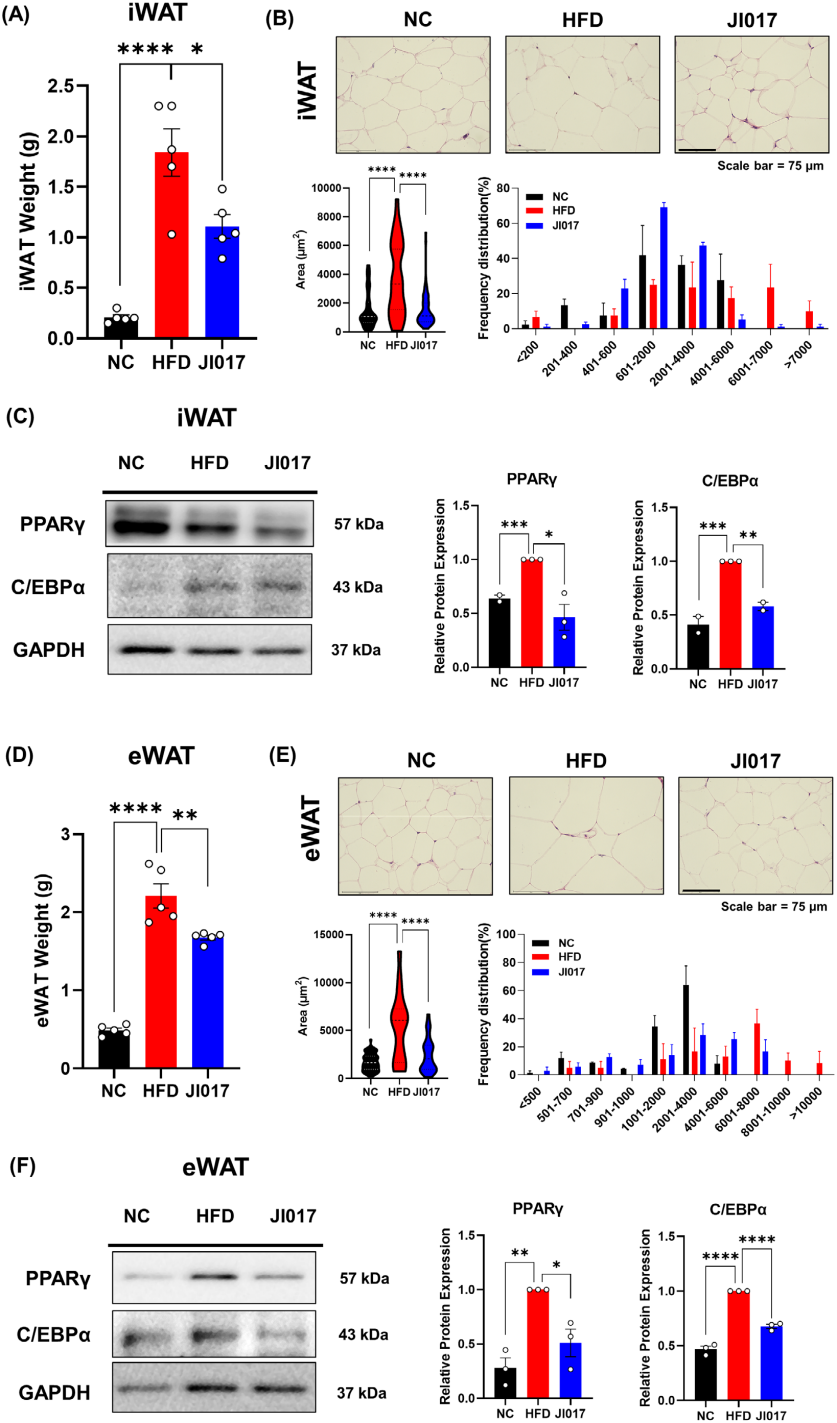
Effect of JI017 on WAT adipogenesis in HFD‐fed C57BL/6J mice. (A) iWAT weight was measured. (B) Histological analysis was performed using H&E staining, and lipid droplet size was measured. (C) Western blot assays were performed in the iWAT of the mice to verify changes in adipogenesis markers. (D) eWAT weight was measured. (E) Histological analysis was performed using H&E staining, and lipid droplet size was measured. (F) Western blot assays were performed in the eWAT of the mice to verify changes in adipogenesis markers. Significant differences between the groups were assessed using one‐way ANOVA with Tukey's multiple comparison test. Data are expressed as the mean ± SEM of three or more independent experiments. *p < 0.05, **p < 0.01, ***p < 0.001, ****p < 0.0001. NC, normal control group; HFD, high‐fat diet group; JI017, HFD diet plus JI017 group.

**FIGURE 4:**
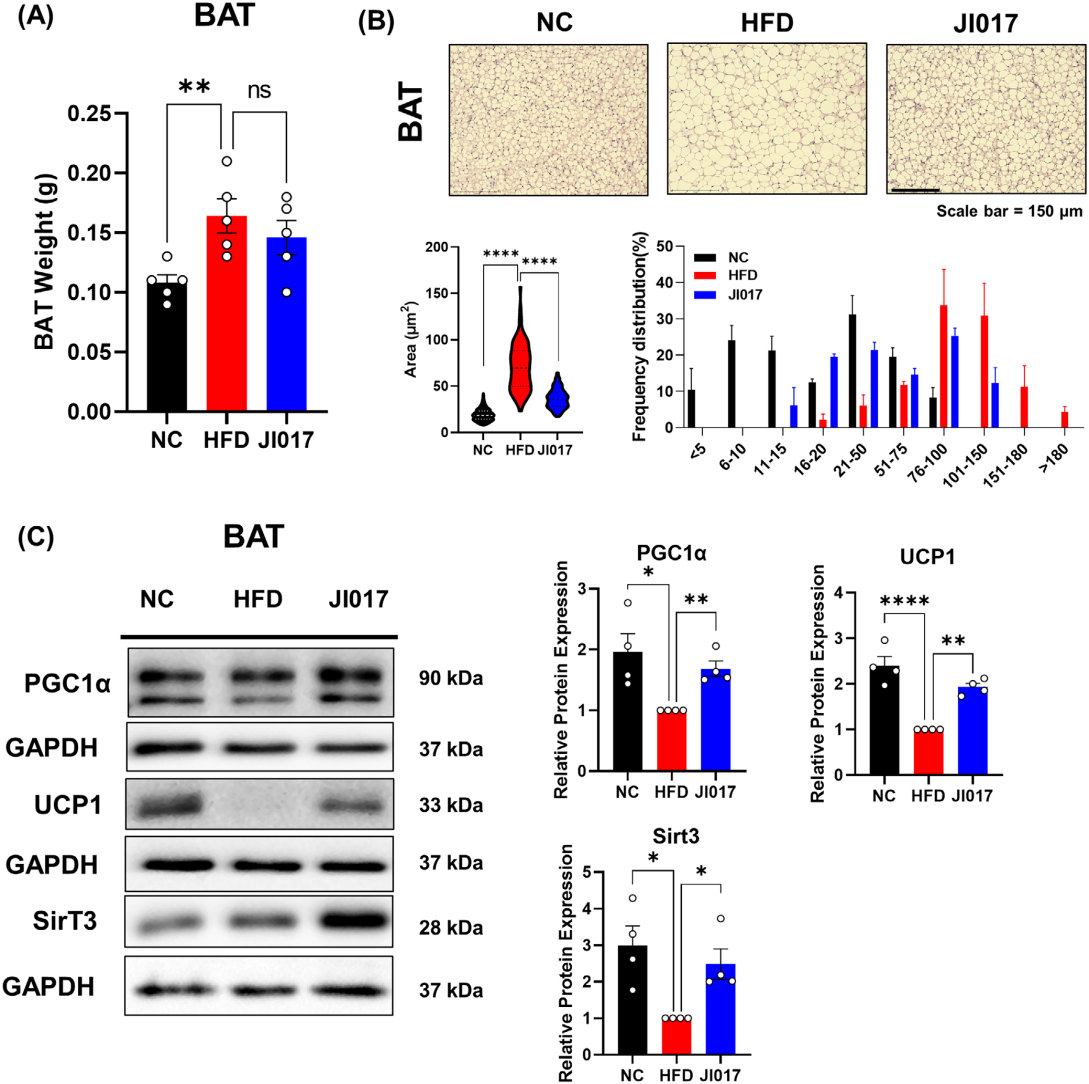
Effect of JI017 on BAT thermogenesis in HFD‐fed C57BL/6J mice. (A) BAT weight was measured. (B) Histological analysis was performed using H&E staining and lipid droplet size was measured. (C) Western blot assays were performed in the BAT of the mice to verify changes in thermogenesis markers. Significant differences between the groups were assessed using one‐way ANOVA with Tukey's multiple comparison test. Data are expressed as the mean ± SEM of three or more independent experiments. *p < 0.05, **p < 0.01, ***p < 0.001, ****p < 0.0001. NC, normal control group; HFD, high‐fat diet group; JI017, HFD diet plus JI017 group.

iWAT and eWAT store excessive energy in the form of lipids (Luo and Liu 2016), which is a typical hallmark of obesity and is associated with metabolic diseases such as NAFLD (Lee et al. 2023). The weights of both iWAT and eWAT were significantly lower in the JI017‐treated mice compared to controls (Figure 3A,D). H&E staining revealed a notable reduction in adipocyte size in both WAT depots (Figure 3B,E), indicating decreased fat storage. Western blot analysis of both depots demonstrated decreased expression of PPARγ and C/EBPα (Figure 3C,F), key transcription factors in adipogenesis (Madsen et al. 2014). BAT plays a central role in whole‐body energy metabolism (Moonen et al. 2019), and low BAT activity is closely linked to NAFLD (Ahmed et al. 2021). Therefore, we investigated BAT responses to JI017 treatment. While no difference in BAT mass was observed (Figure 4A), histological examination showed a marked reduction in lipid droplet size within the BAT (Figure 4B), consistent with increased fat utilization. Western blot analysis (Figure 4C) revealed increased expression of key thermogenic proteins, including UCP1 (Fedorenko et al. 2012), PGC1α (Uldry et al. 2006), and SIRT3 (Sebaa et al. 2019), which are crucial for mitochondrial biogenesis and energy expenditure. These findings suggest that JI017 promotes mitochondrial function and enhances thermogenesis in BAT, contributing to increased energy expenditure and fat oxidation.

### 3.4 JI017 Targets Intersect With NAFLD‐Related Genes and Modulate Oxidative and Metabolic Pathways Through NRF2 Binding and Activation

**FIGURE 5:**
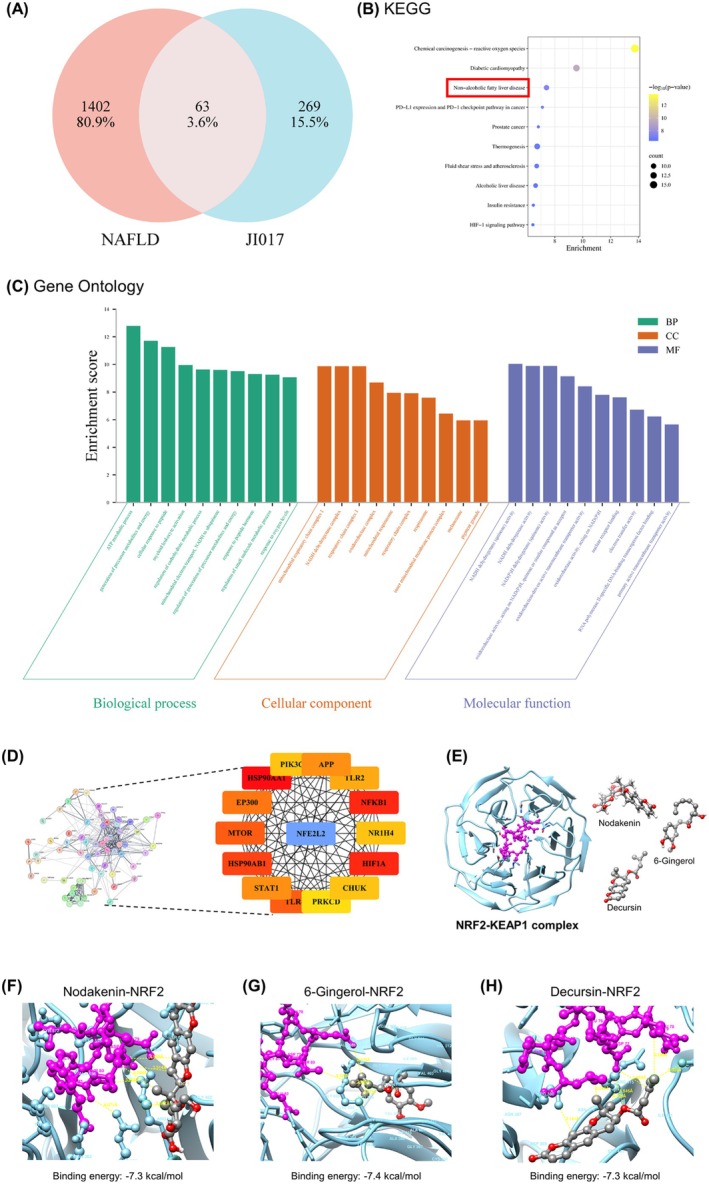
In silico analysis of the effect of JI017 on NAFLD by NRF2 as a potential target. (A) Network pharmacology analysis determines 63 shared genes related to JI017 and NAFLD. (B) KEGG analysis, (C) GO analysis, and (D) PPI network of the common genes. (E) Protein structure of NRF2 and molecular structures of nodakenin, 6‐gingerol, and decursin. Molecular docking analysis of NRF2 with (F) nodakenin, (G) 6‐gingerol, and (H) decursin.

The liver is another organ centrally linked to metabolic dysfunctions (Do et al. 2024). To evaluate the potential relevance of JI017 to NAFLD, we performed a target overlap analysis between JI017‐associated genes and NAFLD‐related gene signatures and identified 63 shared genes (Figure 5A), suggesting a potential mechanistic intersection. Pathway enrichment analysis using KEGG revealed that JI017‐associated targets were significantly enriched in multiple NAFLD‐relevant pathways, including reactive oxygen species (ROS), diabetic cardiomyopathy, alcoholic liver disease, thermogenesis, insulin resistance, and NAFLD itself (Figure 5B). Together, these findings implicate JI017 as a potential modulator of NAFLD pathology through coordinated targeting of mitochondrial function, ROS metabolism, and insulin signaling pathways. To systematically characterize the biological functions of JI017‐associated genes, we performed GO enrichment analysis across BP, CC, and MF. Among BP terms, the most significantly enriched processes included ATP metabolic process, generation of energy, mitochondrial electron transport, and response to oxygen levels, suggesting the potential effect of JI017 on energy metabolism (Figure 5C, green). In the CC category, top‐ranking terms were highly mitochondrial‐centric, including the mitochondrial inner membrane, respiratory chain complex, and proton‐transporting ATP synthase complex (Figure 5C, orange). For MF, enriched functions involved NADH dehydrogenase activity, oxidoreductase activity, and proton transmembrane transporter activity (Figure 5C, blue), implicating redox regulation and mitochondrial electron transport as likely mechanisms of action. Together, these GO signatures provide a mechanistic rationale linking JI017 target genes to mitochondrial function and redox homeostasis, which are processes commonly dysregulated in obesity and NAFLD.

To further investigate the mechanistic pathway of JI017, we constructed a PPI network from the shared gene set of JI017 and NAFLD. Network topology analysis using degree centrality identified NFE2L2, the gene for nuclear factor erythroid 2‐related factor 2 (NRF2) protein which is a master regulator of oxidative stress responses (Ma 2013), as a prominent hub node (Figure 5D). Other highly connected nodes included PIK3CA, HSP90AA1, MTOR, an d STAT1, suggesting regulatory convergence on pathways related to inflammation, metabolism, and cellular stress responses. In response to oxidative stress such as excessive lipid peroxidation, NRF2 dissociates from its negative regulator KEAP1 and translocates to the nucleus, where it activates antioxidant genes (Dodson et al. 2019; Kansanen et al. 2013). Therefore, ligand interactions that stabilize NRF2 and prevent formation of the NRF2‐KEAP1 complex could be a direct target of JI017. Based on the structure of this complex and the components identified by HPLC (Figure 5E), we next performed a molecular docking simulation to see whether NRF2 can be a direct target of JI017. The constituents of JI017, nodakenin (Figure 5F), 6‐gingerol (Figure 5G), and decursin (Figure 5H) were docked at the NRF2 interface, each exhibiting stable interactions that included hydrogen bonds and hydrophobic contacts. Notably, nodakenin formed extensive interactions with the Ser^508^ and Arg^483^ residues, which are implicated in NRF2 activation. 6‐gingerol and decursin also bound to Ser^508^ and Arg^483^ residues, respectively (Table 1). These findings position NRF2 as a mechanistic nexus in the pharmacological action of JI017. Together, systems‐level network analysis and structure‐based docking support the therapeutic potential of JI017 in modulating redox‐sensitive metabolic pathways in NAFLD.

**TABLE 1:** Information from molecular docking analysis.

Molecule	Binding energy (kcal/mol)	Binding amino acid residues
5WFV‐Nodakenin	−7.3	Chain A: ARG380 ASN387 THR388 ASP389 ASN414 ARG415 SER431 HIS432 GLY433 CYS434 HIS436 ILE461 PHE478 GLY480 THR481 ASN482 ARG483 SER508 TYR525 GLY527 GLN528 Chain P: LEU76 ASP77 GLU78 GLU79 LEU84
5WFV‐ 6‐Gingerol	−7.4	Chain A: GLY364 LEU365 ALA366 GLY367 CYS368 ARG415 ILE416 GLY417 VAL418 GLY419 ILE461 GLY462 VAL463 GLY464 VAL465 ALA466 VAL467 SER508 GLY509 ALA510 GLY511 VAL512 CYS513 VAL514 SER555 ALA556 LEU557 GLY558 ILE559 THR560 VAL561 GLY603 VAL604 GLY605 VAL606 ALA607 Chain P: GLU79 THR80
5WFV‐Decursin	−7.3	Chain A: ARG380 ASN387 ASP389 ASN414 ARG415 SER431 HIS432 GLY433 CYS434 HIS436 ILE461 PHE478 GLY480 THR481 ARG483 TYR525 GLY527 Chain P: LEU76 ASP77 GLU78 GLU79 PHE83 LEU84

### 3.5 JI017 Reduces Lipid Accumulation in the Liver of HFD‐Fed C57BL/6J Mice Through Activation of the Antioxidant NRF2‐HO‐1 Axis

**FIGURE 6:**
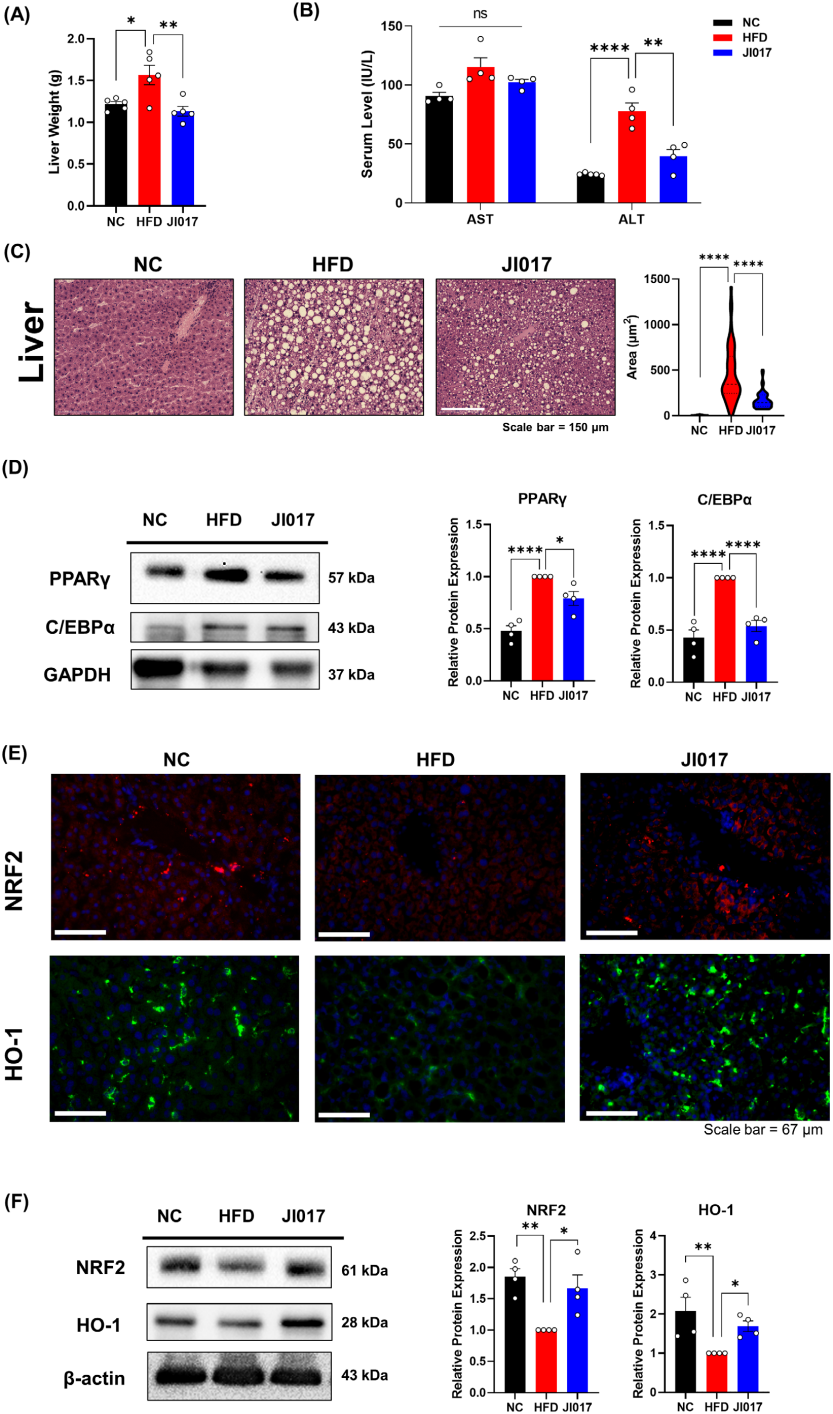
Effect of JI017 on liver adipogenesis and oxidative stress in HFD‐fed C57BL/6J mice. (A) Liver weight was measured. (B) Serum levels of AST and ALT were measured. (C) Histological analysis was performed using H&E staining. (D) Western blot assays were performed in the liver of the mice to verify changes in adipogenesis markers. (E) IF staining and (F) western blot assays of NRF2 and HO‐1 were performed in liver tissues. Significant differences between the groups were assessed using one‐way ANOVA with Tukey's multiple comparison test. Data are expressed as the mean ± SEM of three or more independent experiments. *p < 0.05, **p < 0.01, ***p < 0.001, ****p < 0.0001. NC, normal control group; HFD, high‐fat diet group; JI017, HFD diet plus JI017 group.

Based on these in silico findings, we investigated the effect of JI017 on the most evident NAFLD symptom, lipid accumulation in the liver. JI017‐treated mice showed a significant reduction in liver weight compared to HFD‐fed controls (Figure 6A). Histological analysis of the liver using H&E staining revealed reduced lipid accumulation (Figure 6B), suggesting that JI017 may protect against obesity‐induced fatty liver. Furthermore, serum markers of liver injury, AST and ALT, were significantly lower in the JI017 group (Figure 6C), indicating that JI017 treatment protects against obesity‐induced liver injury. Western blot analysis showed decreased expression of PPARγ and C/EBPα (Figure 6D), which are key regulators of adipogenesis and hepatic lipid synthesis (Lee et al. 2018). These results suggest that JI017 effectively inhibits fat accumulation via suppression of adipocyte differentiation, likely contributing to the observed improvement in obesity‐related parameters.

**FIGURE 7:**
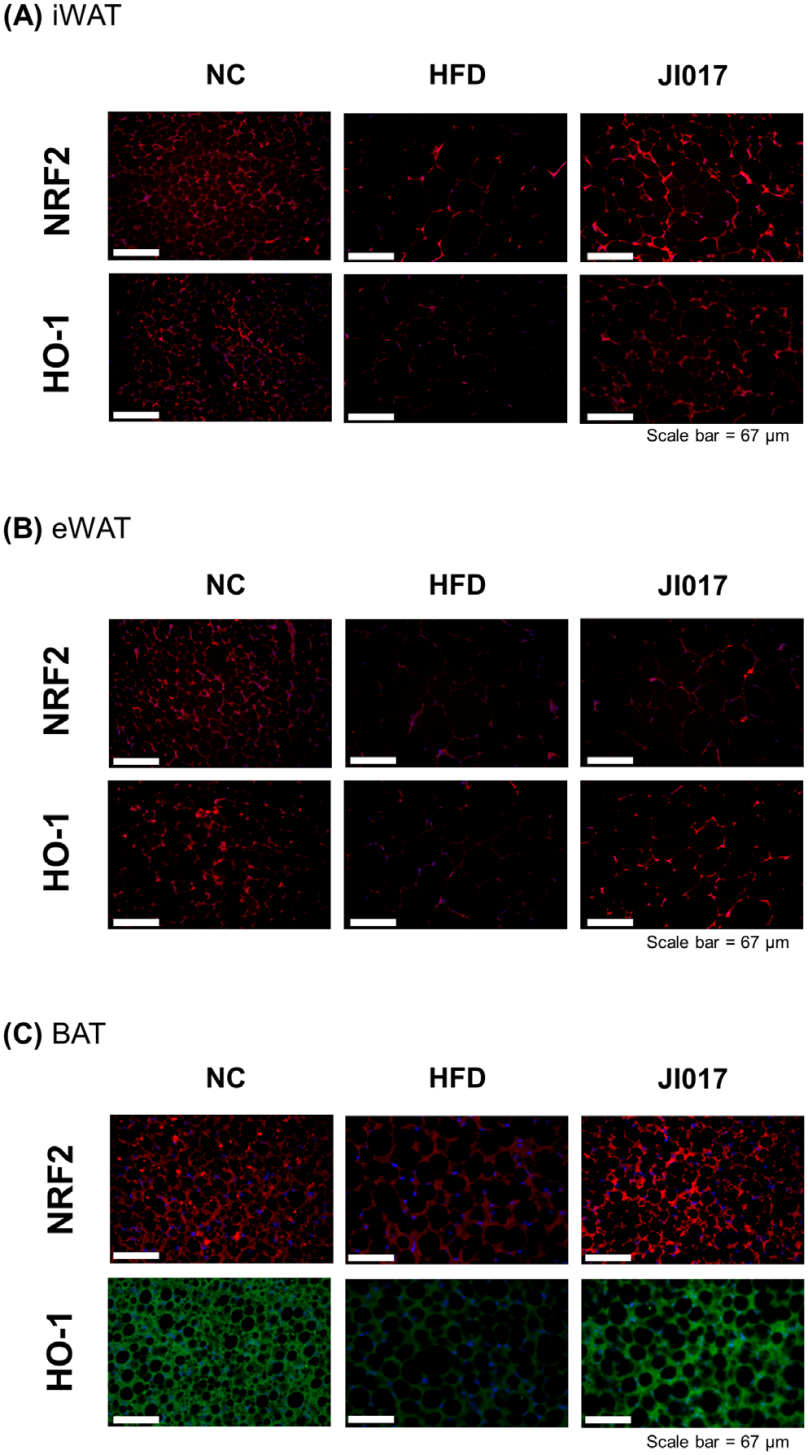
Effect of JI017 on the antioxidant pathway in adipose tissues of HFD‐fed C57BL/6J mice. IF staining of NRF2 and HO‐1 in (A) iWAT, (B) eWAT, and (C) BAT of the mice was performed. NC, normal control group; HFD, high‐fat diet group; JI017, HFD diet plus JI017 group.

Oxidative stress arises when the production of ROS exceeds the capacity of the body's antioxidant systems. This imbalance damages cellular macromolecules such as lipids, proteins, and DNA, contributing to aging and a variety of pathological conditions including cancer, neurodegeneration, and cardiovascular diseases (Ma 2013). Excessive fat accumulation in obesity results in low‐grade inflammation from local and systemic production of pro‐inflammatory cytokines (Sengenès et al. 2007), and this increases ROS (Rzheshevsky 2013). The body's defense system in turn activates antioxidant pathways. Additionally to NRF2, the potential therapeutic target of JI017, heme oxygenase‐1 (HO‐1) together with NRF2 consists the key antioxidant pathways (Loboda et al. 2016). Moreover, their involvement in obesity and NAFLD is also well‐known (McClung et al. 2022; Vasileva et al. 2020). The expression levels of NRF2 and HO‐1 were notably increased in the liver of HFD‐fed mice following JI017 treatment (Figure 6F,G). Similarly, we observed that these two proteins are decreased by HFD in BAT, iWAT, and eWAT, whereas JI017 treatment markedly increased their expressions (Figure 7A–C). These findings indicate that JI017 promotes lipid metabolism while enhancing the antioxidant capacity, potentially protecting against oxidative stress and thereby alleviating hepatic steatosis.

### 3.6 JI017 Modifies the Composition of the Intestinal Microbiome

**FIGURE 8:**
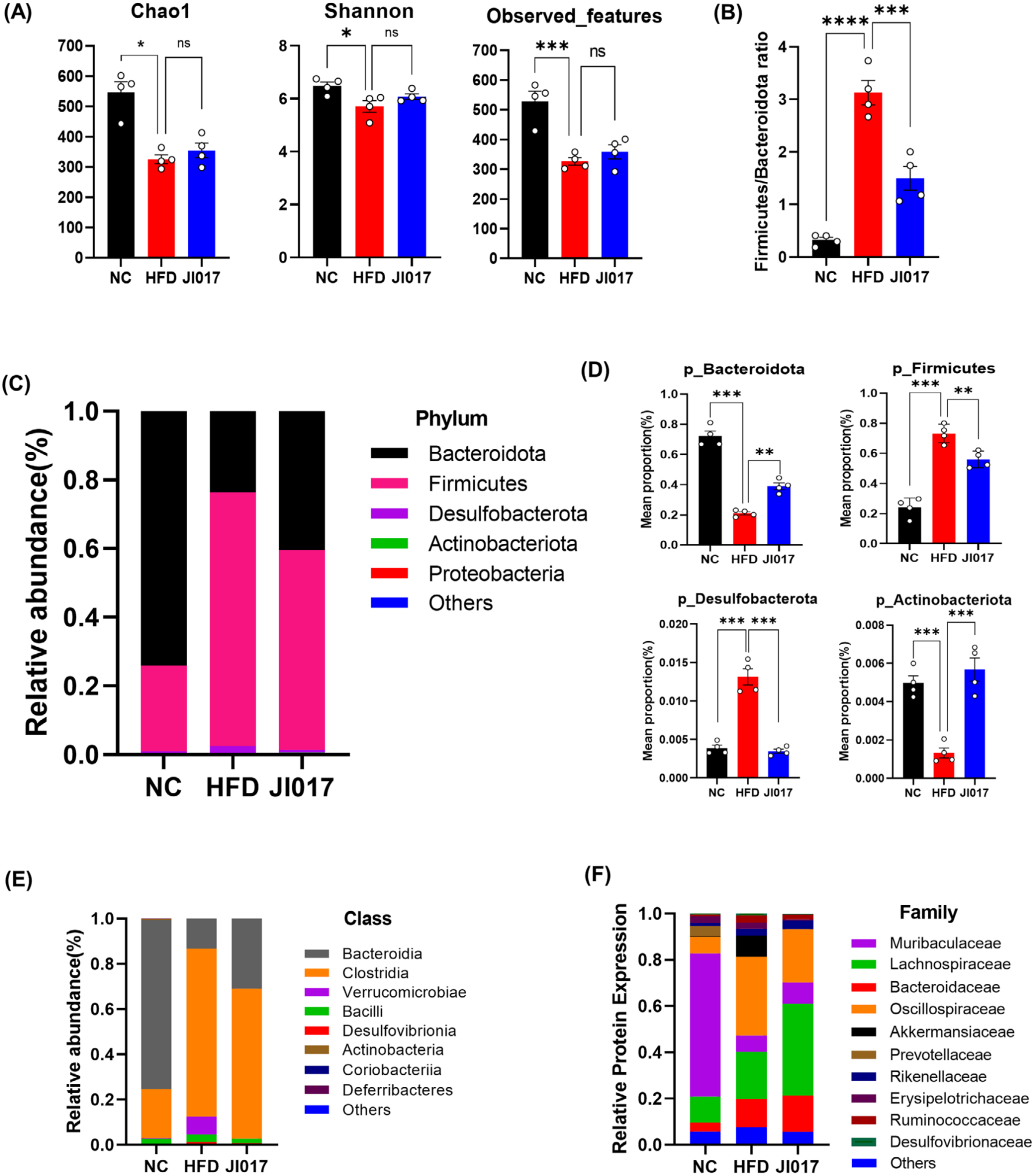
Effect of JI017 on the gut microbiome in HFD‐induced obese C57BL/6J mice. (A) Alpha diversity analysis of the gut microbiota in feces. Chao 1 index, Shannon index, and observed features index of the gut microbiota. (B) The proportion of *Firmicutes* and *Bacteroidetes* in the gut microbiota of NC, HFD and JI017. Relative abundances of gut microbiota at the (C, D) Phylum, (E) Class, and (F) Family levels. Data are expressed as the mean ± SEM of three or more independent experiments. NC, normal control group; HFD, high‐fat diet group; JI017, HFD diet plus JI017 group.

Recent studies have shown that gut microbiota may affect the host's ability to store energy (Bäckhed et al. 2004). Moreover, the gut microbiota influences oxidative stress through the synthesis of metabolites, regulation of antioxidant enzymes, and the maintenance of gut homeostasis (Ballard and Towarnicki 2020). NRF2 and HO‐1 signaling pathways play a critical role in maintaining the homeostasis of gut microbiota. Dysregulation of these pathways has been associated with metabolic disorders, including obesity, through their effects on oxidative stress, inflammation, and barrier function (Funes et al. 2020; O'Rourke et al. 2024). NRF2 activation influences the composition of the gut microbiota by regulating antioxidant defense mechanisms and improving gut barrier integrity (Liu et al. 2024). Similarly, HO‐1 induction exerts anti‐inflammatory effects and modulates the microbial community, contributing to gut and systemic health (Sebastián et al. 2018). These facts suggest the potential of the gut microbiome as a therapeutic target for obesity treatment. Three types of analyses were conducted to characterize microbial richness (Chao1 and observed features) and diversity (Shannon). JI017 treatment did not significantly affect microbial richness or diversity relative to the control group (Figure 8A). To further explore gut microbiome alterations, histograms were generated to display the species composition of the gut microbiota community. At the phylum level, the gut microbiota of fecal samples from all three groups was primarily composed of *Bacteroidetes* and *Firmicutes*. The relative abundance of *Bacteroidetes* increased, while *Firmicutes* decreased, leading to a significant decrease of *the Firmicutes/Bacteroidetes* ratio by JI017 treatment (Figure 8B,C). Additionally, the relative abundance of *Desulfobacterota* in the feces of JI017‐treated mice was lower than in the control group, whereas the relative abundance of *Actinobacteriota* was higher (Figure 8D). *Bacteroidia*, a member of the *Bacteroidota* class, is associated with a lean phenotype and may play a protective role against obesity. Studies have demonstrated that increased levels of *Bacteroidota*, including *Bacteroidia*, are correlated with reduced body fat and improved metabolic health (Amabebe et al. 2020). Conversely, reduced levels of *Clostridia* may increase the susceptibility of the gut to inflammation, which is often linked to the development of metabolic disorders and obesity (Vallianou et al. 2020). At the class level, the relative abundances of *Bacteroidia* and *Clostridia* in the gut microbiota of JI017‐treated mice were higher compared to the control group (Figure 8E), while at the family level, the relative abundances of *Muribaculaceae*, *Lachnospiraceae*, and *Bacteroidaceae* were higher (Figure 8F). These results suggest that JI017 may help prevent obesity and associated NAFLD and simultaneously promote the improvement of metabolic disorders by inducing body fat reduction, enhancing energy metabolism, and exerting anti‐inflammatory effects via beneficial shifts in the intestinal microbiota.

## 4 Discussion

The results of this study demonstrate the significant potential of the multi‐herbal decoction JI017 in alleviating HFD‐induced obesity in C57BL/6J mice. Our findings suggest that JI017 improved glucose metabolism, as evidenced by enhanced glucose tolerance, and exerted profound anti‐obesity effects by inhibiting WAT adipogenesis, activating BAT thermogenesis, and alleviating hepatic oxidative stress. These multi‐targeted effects suggest the promise of JI017 as a comprehensive therapeutic approach for NAFLD management.

JI017 is composed of three herbs; *Angelica gigas*, *Zingiber officinale* , and processed *Aconitum carmichaeli*, in a 2:1:1 ratio. Originally designed to treat various types of cancers (Kim et al. 2021; Kim and Ko 2021; Ku et al. 2023), our drug‐repositioning strategy shows that it also has beneficial effects on metabolic diseases. *Zingiber officinale* is a widely used herb to treat obesity and related complications. Research has shown that *Z. officinale* shows protective effects against gold‐thioglucose‐ (Goyal and Kadnur 2006) and HFD‐ (Ebrahimzadeh Attari et al. 2015; Kim et al. 2018; Lee et al. 2024; Misawa et al. 2015; Seo et al. 2021; Wang et al. 2019) induced metabolic syndromes. Its effect has also been demonstrated in clinical studies (Ebrahimzadeh Attari et al. 2016; Ghoreishi et al. 2024; Maharlouei et al. 2019; Salih et al. 2023). *Aconitum carmichaeli* is toxic (Chan et al. 2021), therefore we processed *A. carmichaeli* to remove its toxic component aconitine (Gao et al. 2022). Anti‐obesity effects of Aconitum have also been reported, mostly by animal studies (Su et al. 2020; Subash and Augustine 2012). Previous studies demonstrated that components of *Angelica gigas* such as angelan, decursin, decursinol, and nodakenin exert anti‐obesity effects (Hwang et al. 2012; Jin et al. 2021; Kim et al. 2008; Park et al. 2019). Based on these clues, we expected that JI017 may show beneficial effects on obesity and NAFLD, and thus conducted this study. Here, we show that standardized JI017 modulates multiple NRF2‐centered pathways, concurrently suppressing hepatic and adipose lipid accumulation through decreased lipogenesis, inducing BAT thermogenic programming, and fortifying antioxidant defenses, thereby resulting in greater reductions in steatosis and weight gain than would be anticipated from isolated pathways.

PPARγ and C/EBPα are crucial in regulating fat cell development and metabolism in obesity (Madsen et al. 2014). PPARγ is a key transcription factor that promotes adipogenesis, helping to convert preadipocytes into mature adipocytes, and regulating lipid storage and insulin sensitivity. C/EBPα works alongside PPARγ to drive the differentiation of adipocytes and maintain their function (Rosen et al. 2002). Both factors are vital in the development of obesity, as their dysregulation can lead to excessive fat accumulation, impaired insulin sensitivity, and metabolic disturbances (Ahmadian et al. 2013). One of the key findings of this study is the reduction in iWAT and eWAT mass following treatment with the herbal decoction. This reduction is likely mediated by the suppression of PPARγ and C/EBPα, transcription factors that govern adipogenesis. By inhibiting these pathways, the herbal extract effectively reduced the differentiation of preadipocytes into mature adipocytes, thereby limiting the expansion of WATs.

In addition to its effects on WATs, JI017 significantly increased BAT thermogenesis. BAT is known for its role in dissipating energy as heat through a process called uncoupled respiration (Zhang et al. 2021). UCP1 and PGC‐1α are key mediators of thermogenic function of BAT. UCP1 is essential for generating heat through non‐shivering thermogenesis by dissipating energy as heat rather than storing it as fat (Fedorenko et al. 2012). PGC‐1α acts as a coactivator that enhances the expression of UCP1 and other genes involved in mitochondrial biogenesis and energy metabolism in BAT (Uldry et al. 2006). Increased activity of PGC‐1α and UCP1 in BAT is associated with improved metabolic rate and reduced fat accumulation, making them important targets for strategies to treat obesity (Lidell et al. 2014). The promotion of BAT activity by JI017 administration suggests a novel mechanism by which it mitigates obesity, further highlighting the potential of herbal medicine to modulate energy balance in favor of weight reduction.

Moreover, JI017 showed a protective effect against oxidative stress. Excessive oxidative stress is a common consequence of obesity (Milagro et al. 2006), and a significant contributor to the progression of metabolic diseases such as NAFLD (Furukawa et al. 2004). HO‐1 and NRF2 are pivotal for body metabolism and health by managing oxidative stress and inflammation (Galicia‐Moreno et al. 2020; Ryter 2022). HO‐1 helps break down heme, producing antioxidant biliverdin and regulating iron levels, thus protecting the liver from damage (Canesin et al. 2022; Yu et al. 2010). NRF2, on the other hand, activates various antioxidant and detoxification pathways, including the expression of HO‐1 (Ma 2013). Together, they synergistically contribute to liver protection, reduce inflammation, and promote overall liver function, making them critical for maintaining liver health and responding to liver diseases (Fuertes‐Agudo et al. 2023; Yuan et al. 2023; Yun et al. 2010). Also, oxidative stress is linked to obesity (Furukawa et al. 2004), mainly due to its impact on adipogenesis (Fernando et al. 2020). Notably, HO‐1 and NRF2 are both involved in the adipogenesis process (Annie‐Mathew et al. 2021; Bellner et al. 2020). We found that NRF2 is a potential target of JI017 in the context of NAFLD based on in silico analysis. Particularly, the active compounds of JI017, including nodakenin, 6‐gingerol, and decursin, showed potentially stable binding to NRF2, preventing formation of the NRF2‐KEAP1 complex and thereby inducing the antioxidant program through NRF2 activation. From in vivo data, we showed that the increased expression of NRF2 and HO‐1 indicates the ability of JI017 to enhance the oxidative defense system not only in the liver, but also in other organs related to metabolism such as BAT and WATs. This reduction in oxidative stress is important, as it not only protects these organs from oxidative damage, but also helps maintain overall metabolic homeostasis.

JI017 is an herbal formula combining three herbs: *A. gigas* , *Z. officinale* , and *A. carmichaeli*. Each herb exhibits distinct modulatory effects on gut microbial communities. Nodakenin, a bioactive compound of *A. gigas* , alleviates obesity‐ and colitis‐associated cancer through gut microbiota modulation (Chung et al. 2025). *Lactiplantibacillus plantarum* SKO‐001, is a probiotic strain isolated from *A. gigas* , which shows body fat‐reducing effects in a 12‐week double‐blind, randomized clinical trial of 100 participants (Shin et al. 2024). Although not directly a gut microbiome study, given the close link between the gut microbial community and intestinal health and barrier integrity (Ghosh et al. 2021), reports on the protective effect of decursin and *A. gigas* on dextran sodium sulfate (DSS)‐induced colitis (Oh et al. 2017; Wang et al. 2025) support such fact. 6‐Gingerol, a major bioactive compound of *Z. officinale* , significantly alters gut microbial composition in high‐fat diet‐induced obese mouse models by increasing *Proteobacteria* and decreasing *Bacteroidetes*, while enriching weight loss‐associated taxa such as *Akkermansia* and *Muribaculaceae*, and reducing weight gain‐associated taxa like *Lactobacillus reuteri* and *Lachnospiraceae* (Alhamoud et al. 2023). *Z. officinale* extract itself also has microbiome‐regulating effects (Kim et al. 2025). Polysaccharides isolated from *A. carmichaeli* modulate gut communities including *Bacteroides*, *Dubosiella*, *Alistipes*, and *Prevotella*, restore short‐chain fatty acid production, and exhibit anti‐inflammatory effects via inhibition of NOD1 and TLR4 pathways, improving gut barrier integrity in DSS‐induced colitis models (Fu et al. 2022). These distinct mechanisms, including modulation of microbial flora, phylum‐level community shifts, and immune‐inflammatory regulation, collectively provide biological plausibility for the observed changes in gut microbiome following treatment with JI017. This is likely via effects on the gut‐liver axis, metabolic pathways, and host immune responses.

NRF2 and HO‐1 signaling pathways are closely linked to gut microbiota (Funes et al. 2020; O'Rourke et al. 2024), therefore our study led on to gut microbiome analysis. In particular, when investigating the role of gut microbiota in obesity, analyzing the relative abundance of *Bacteroidetes* and *Firmicutes* can provide valuable insights (Magne et al. 2020). The abundance of *Firmicutes* increases significantly in obesity (Koliada et al. 2017), as Firmicutes are efficient at fermenting dietary fibers into additional energy sources, contributing to excessive caloric harvest and obesity (Turnbaugh et al. 2006). *Bacteroidetes* is associated with a lean phenotype and may play a protective role against obesity (Amabebe et al. 2020). Therefore, an elevated *Firmicutes*/*Bacteroidetes* ratio is widely accepted as a hallmark of gut microbial dysbiosis in obesity, consistently observed across human patients and animal models (Ley et al. 2005, 2006). JI017 significantly decreased this ratio, suggesting its impact on the gut microbiome contributes to its anti‐obesity effect. Elevated levels of bacteria Class *Desulfovibrionia* are associated with obesity and metabolic disorders. Increases in these microbes may impair nutrient absorption and energy balance, leading to weight gain (Ramadan et al. 2022). JI017 treatment suppressed HFD‐induced increase in *Desulfovibrionia* abundance. At the family level, increased abundance of *Muribaculaceae* has been associated with the beneficial effects of a plant‐based diet on inflammatory bowel disease, obesity, and type 2 diabetes (Zhu et al. 2024), and we observed higher *Muribaculaceae* abundance in the JI017 group. The relative abundance of *Clostridium* and *Clostridiales* was significantly reduced in db/db mice compared to WT mice, and oral administration of *Clostridium butyricum* restored the protective microbiota through activation of the NRF2/HO‐1 pathway (Zhou et al. 2024). JI017 treatment also increased the abundance of *Clostridia*. Additionally, an associated increase in *Lachnospiraceae* in the JI017 treatment group was seen, as *Lachnospiraceae*, *Oscillospiraceae*, *Acutalibacteraceae*, and *Ruminococcaceae* are identified as dominant families in the gut microbiota, comprising 58% of *Clostridia* species (Atarashi et al. 2013). Overall, our results show that JI017 successfully reverses the increased *Firmicutes*/*Bacteroidetes* ratio and other changes induced by HFD administration, suggesting that its anti‐obesity effects involve the restoration of gut microbiome balance. However, direct mechanistic validation of the full JI017 formula remains necessary. These findings should be interpreted with caution as correlation does not imply causation. The gut microbial shifts may also be secondary to weight loss. Potential mechanisms linking *Firmicutes*/*Bacteroidetes* ratio changes to metabolic improvement include altered SCFA production profiles and improved gut barrier function reducing metabolic endotoxemia caused by LPS (Cani et al. 2007; den Besten et al. 2013). Therefore, future investigations employing antibiotic depletion, and metabolomics profiling are planned to establish definitive relationships between JI017 and gut microbiota. In our next study, we plan to perform experiments involving antibiotic‐cocktail depletion of the gut microbiome in mice to test whether the effect of JI017 persists in microbiota‐depleted animals, establishing whether gut microbiota are necessary mediators. Also, precise metabolomics study focusing on the fatty acid profiles may provide necessary information on the role of the gut microbiome. These approaches will be critical to clarify the mechanistic relevance of the observed correlations and are acknowledged as a limitation of the current study.

## 5 Conclusion

In conclusion, our findings collectively suggest that JI017 ameliorates NAFLD through multiple, interrelated pathways. By suppressing adipogenesis, enhancing non‐shivering thermogenesis, and reducing oxidative stress, JI017 offers a holistic therapeutic approach as a novel treatment for obesity and associated complications such as NAFLD, as shown in this study. This multi‐targeted action is particularly advantageous in the context of NAFLD, which is a condition characterized by complex and multifactorial pathophysiology. The results of this study contribute to the growing body of evidence supporting the use of herbal medicine in the treatment of NAFLD and other obesity‐related metabolic diseases. Given the challenges associated with conventional therapies, such as side effects and limited efficacy, herbal medicine presents a promising alternative or complementary approach. However, further research is necessary to fully elucidate the molecular mechanisms underlying the effects observed in this study. The translational relevance of these findings should also be established to support clinical application. Overall, JI017, the multi‐herbal decoction studied here, offers a potential therapeutic strategy for NAFLD.

## Author Contributions

**Harim Kim:** writing – original draft, investigation, data curation. **Hye‐Lin Kim:** writing – original draft, investigation, data curation, funding acquisition. **Mina Boo:** investigation. **Hyunyoung Choi:** investigation. **Jaehyun Han:** investigation. **Seunghyun Nam:** investigation. **So Jung Kang:** writing – original draft. **Jae Kyeom Kim:** methodology. **Yohan Han:** methodology. **Ji Hoon Jung:** methodology. **Kwan‐Il Kim:** methodology. **Woojin Kim:** methodology. **Jae‐Young Um:** methodology. **Jinbong Park:** writing – review and editing, conceptualization, funding acquisition, supervision, project administration. **Leo E. Otterbein:** writing – review and editing, methodology, supervision. **Hyo In Kim:** writing – original draft, writing – review and editing, investigation, methodology, supervision. **Seong‐Gyu Ko:** writing – review and editing, conceptualization, funding acquisition, supervision. All data were generated in‐house, and no paper mill was used. All authors agree to be accountable for all aspects of work ensuring integrity and accuracy.

## Conflicts of Interest

The authors declare no conflicts of interest.

## Data Availability

The data that support the findings of this study are available from the corresponding author upon reasonable request.
